# Global and regional inequalities in dairy recommendations: a natural language processing analysis of food-based dietary guidelines across income groups

**DOI:** 10.1016/j.lana.2026.101550

**Published:** 2026-07-06

**Authors:** Ayleen Bertini, Hugo Cáceres-Ozimica, Rodrigo Valenzuela, Samuel Durán-Agüero

**Affiliations:** a Facultad de Odontología, Universidad San Sebastián, Santiago, Chile; b Facultad de Ciencias de la Rehabilitación y Calidad de Vida, Universidad San Sebastián, Chile; c Department of Nutrition, Faculty of Medicine, University of Chile, Santiago, Chile

**Keywords:** Dietary guidelines, Dairy products, Public nutrition, Human development index, Natural language processing

## Abstract

**Background:**

Food-based dietary guidelines (FBDGs) are key public health instruments aimed at promoting healthy dietary patterns. However, dairy-products recommendations vary substantially across countries, reflecting not only scientific evidence but also socioeconomic conditions, institutional capacity, and food system characteristics. The extent to which these differences are structured linguistically across income levels has not been systematically quantified. The present study aims to explore the use of advanced NLP techniques to characterize semantic differences and similarities in dairy-products dietary messages within FBDGs from countries with different levels of socioeconomic development.

**Methods:**

We conducted a comparative analysis of dairy-products recommendations extracted from national FBDGs officially recognised by the Food and Agriculture Organization, covering 98 countries. Using advanced natural language processing techniques, including lexical frequency analysis, co-occurrence networks, and latent topic modelling, we examined semantic patterns in recommendation statements and justificatory texts. Countries were stratified according to World Bank income group classifications.

**Findings:**

Across all income groups, “milk” emerged as the central lexical anchor of dairy recommendations. However, high-income countries demonstrated greater lexical diversity and semantic complexity, incorporating differentiated references to product types, fat content, and fermentation (e.g., yogurt, cheese, low-fat). In contrast, low- and lower-middle-income countries presented more general and nutritionally basic messaging, primarily focused on consumption adequacy and child nutrition. Justification texts consistently contained higher nutrient-related terminology density than recommendation statements.

**Interpretation:**

Dairy-products dietary messaging in national FBDGs shows consistent descriptive differences across income groups. These semantic disparities likely reflect contextual differences in institutional capacity, epidemiological priorities, and food system infrastructure. The findings may be particularly relevant for the Americas, where high-, upper-middle-, and lower-middle-income countries coexist within a region undergoing rapid nutrition transitions and facing the persistent triple burden of malnutrition. In this context, PAHO/WHO may play an important role in supporting greater harmonisation of dietary guidance across diverse socioeconomic settings. NLP-based approaches offer scalable tools for monitoring global nutrition policy discourse and supporting evidence-informed policy development.

**Funding:**

Research supported by the Vice-Presidency of Research and Doctoral Studies of Universidad San Sebastián, Grant USS-FIN-26-APCS-01; Institutional collaboration provided by the Scientific Committee of Dairy Products of the Chilean Dairy Consortium (Consorcio Lechero) through the "Gracias a la Leche" program.

**Research in context**

**Evidence before this study**

We searched PubMed, Scopus, and Web of Science for articles published between October 2025 and January 2026 using combinations of the terms “food-based dietary guidelines”, “dairy recommendations”, “nutrition policy”, “global dietary guidelines”, and “natural language processing”. Previous studies have documented substantial cross-country variability in the content and framing of food-based dietary guidelines, particularly regarding animal-source foods and dairy products. However, previous study has systematically applied large-scale natural language processing techniques to systematically characterize by integrating lexical frequency analysis, co-occurrence network analysis, and topic modelling, we provide quantitative evidence of systematic differences in dietary messaging according to income level and human development index categories.

**Added value of this study**

This study is the first to apply natural language processing methods to analyse dairy products dietary messaging across a global corpus of national food-based dietary guidelines. By combining lexical frequency analysis, co-occurrence network analysis, and topic modelling (Latent Dirichlet Allocation), we provide quantitative insights into descriptive differences in dietary messaging according to income level and human development index categories. This approach offers a scalable and reproducible framework for evaluating nutrition policy language beyond traditional qualitative comparisons.

**Implications of all the available evidence**

Our findings suggest that differences in dairy-product dietary guidance reflect broader structural inequalities in institutional capacity, food system development, and public health priorities. Incorporating computational linguistic tools into nutrition policy analysis may support international organizations and national authorities in monitoring guideline coherence, identifying communication gaps, and improving the alignment of dietary recommendations with emerging scientific evidence and global health priorities.

## Introduction

Food-Based Dietary Guidelines (FBDGs) constitute one of the main public policy tools in nutrition, as they aim to promote healthy dietary patterns that are culturally appropriate and aligned with the socioeconomic contexts of each country, aimed at promoting healthy dietary patterns that are culturally appropriate and aligned with the socioeconomic contexts of each country.^1^ These guidelines translate scientific evidence into clear and actionable messages for the general population, influencing both individual dietary behaviours and the design of food programs, public health interventions, and intersectoral policies.^2^

Within FBDGs, recommendations regarding dairy consumption occupy a relevant yet heterogeneous position. Traditionally, dairy products have been promoted for their contribution of essential nutrients such as calcium, high-quality protein, and B-complex vitamins. However, their role in health has been subject to debate, particularly in relation to saturated fat content, lactose intolerance, and their potential contribution to non-communicable chronic diseases.^3^, ^4^, ^5^ Consequently, dietary guidelines show substantial variability in the inclusion of dairy as an independent food group, the recommended frequency of consumption, the types of products encouraged, and the nutritional justifications provided, including considerations related to cancer risk,^6^ obesity^7^ and metabolic health outcomes.^8^^,^^9^

This heterogeneity cannot be explained solely by nutritional evidence. Cultural, economic, productive, and epidemiological factors also influence how dietary recommendations are formulated.^10^, ^11^, ^12^, ^13^ In countries with higher levels of development, dietary guidelines tend to incorporate more specific messages, differentiating between types of dairy products and emphasizing particular nutritional attributes or health considerations. In contrast, in lower-income contexts, recommendations are often more general and primarily focused on meeting basic nutritional needs, especially among vulnerable populations such as children, pregnant women and older individuals. Factors such as food security, local availability, affordability, and environmental sustainability may therefore be weighted differently depending on the socioeconomic context.^11^ Recent evidence highlights that dietary guidelines are increasingly encouraged to incorporate sustainability principles, including plant-forward dietary approaches, the role of fortified plant-based alternatives, and considerations related to nutrient bioavailability, cultural inclusiveness, and environmental impact.^14^

Despite the existence of descriptive and comparative studies examining dietary guidelines, most analyses have relied on traditional qualitative approaches focused on identifying the presence or absence of specific messages or food groups. However, previous evidence suggests that dairy-related messaging within FBDGs encompasses multiple dimensions, including contributions to nutrients of concern (both under- and overconsumption), health outcomes such as bone, cardiometabolic, and immune health, as well as their role within overall dietary patterns.^11^ While these approaches provide valuable insights, they have limitations in capturing semantic complexity, linguistic structure, and underlying discursive patterns, particularly when analysing large volumes of textual information across countries.

Recent advances in artificial intelligence and natural language processing (NLP) provide new opportunities to address these limitations. NLP refers to computational approaches used to process and analyse human language, supporting tasks such as text classification, topic modelling, information extraction, and semantic analysis.^15^ These techniques allow the automated analysis of large text corpora, facilitating the identification of linguistic patterns, thematic emphases, and semantic relationships across documents.^15^

In nutrition research, NLP has been increasingly used to process and analyse complex datasets, including food diaries and dietary records, as well as to integrate information from multiple food composition databases.^16^ In the context of dietary guideline–related research, NLP has also been used to analyse large volumes of public comments submitted during the development process of the Dietary Guidelines for Americans, illustrating its potential to examine textual data linked to nutrition policy processes.^17^ Computational text analysis therefore offers a scalable and objective approach to evaluating the content of dietary guidelines within global nutrition governance frameworks.

However, the use of NLP techniques to comparatively analyse dairy-product recommendations in national dietary guidelines remains limited. Consequently, little is known about how the language used in these documents varies according to countries’ socioeconomic development levels and how such differences may shape the formulation and interpretation of food and nutrition policies.

Computational text analysis offers a scalable and systematic approach to cross-country comparison of policy language, enabling more transparent evaluation of dietary guidelines within global nutrition governance frameworks. Socioeconomic development level was used as an analytical framework because it captures structural differences in institutional capacity, food system complexity, and policy development, which are likely to shape how dietary recommendations are formulated and communicated.

The Americas, particularly Latin America and the Caribbean, represent a region of marked socioeconomic heterogeneity, where countries with high, upper-middle, and lower-middle income levels coexist alongside rapid nutrition transitions and the persistent triple burden of malnutrition.^18^ In this context, understanding how dairy-related dietary messages vary according to development level may contribute to regional discussions on dietary guideline harmonisation and public health nutrition policy promoted by organizations such as PAHO/WHO.^19^

Therefore, the present study aims to explore the use of advanced NLP techniques to characterize semantic differences and similarities in dairy-products dietary messages within FBDGs from countries with different levels of socioeconomic development.

## Methods

### Study design

A cross-sectional, descriptive–analytical study based on documentary analysis and natural language processing of dairy-products recommendations in food-based dietary guidelines. Countries were primarily stratified according to World Bank income group classifications (low, lower-middle, upper-middle, and high income), which served as the main framework for comparative analyses. In addition, the Human Development Index (HDI) from the Human Development Report 2023–2024^20^ was used as a complementary contextual indicator to characterize broader development gradients across countries.

### Food guide message database

Data were collected through a systematic review of each food-based dietary guideline available on the FAO website (n = 98).^2^ The United States was already included among the 98 countries listed in the FAO repository. For this country, the most recent version of the Dietary Guidelines for Americans (2025–2030)^21^ was used to replace the previous version available in the FAO database.^2^ No additional countries were added or excluded. Therefore, the final dataset comprised 98 countries (Supplementary Table S1). No duplicate entries were included, as each country was represented by a single guideline document.

A total of 98 countries were included in the final dataset, and all subsequent NLP analyses were performed on this complete corpus without exclusions. Dairy-products content was identified through a structured manual extraction process. First, each dietary guideline was screened to determine the presence of any recommendation related to dairy products. When such recommendations were identified, the corresponding statements were extracted verbatim. Subsequently, a full-text review of each document was conducted to identify all additional references to dairy products, including both recommendations and their associated justificatory statements. All extracted content was transcribed verbatim into the analytical dataset. To minimize potential bias, the extraction process was reviewed by multiple researchers. All terms present within the extracted dairy-related sections were retained for analysis, including references to other food groups (e.g., meat, fish, eggs), as these form part of the broader dietary context in which dairy recommendations are embedded. The inclusion of these terms was intended to preserve the semantic structure of the texts and ensure accurate representation of how dairy products are framed within overall dietary guidance.

Text preprocessing and lexical analysis were conducted using standardized natural language processing procedures. Lexical frequency was defined as the number of occurrences of each token within the corpus. To ensure comparability across documents of varying length, all frequency-based measures were normalized and expressed as relative frequencies per 1000 tokens. The unit of analysis was defined at the country level, where each national dietary guideline section was treated as a single document. All NLP-derived metrics were interpreted as descriptive indicators of semantic patterns rather than inferential statistical estimates. To ensure comparability across income groups with different corpus sizes, word frequencies were normalized and expressed as relative frequencies per 1000 tokens. Text preprocessing included lowercasing, removal of punctuation and special characters, and exclusion of common English stopwords (e.g., “and”, “the”, “of”). Word clouds were generated based on these normalized frequencies, where word size reflects relative frequency within each income group. This normalization strategy was consistently applied to all frequency-based analyses.

Each country guideline section was treated as a single analytical document within the NLP corpus. In contrast, co-occurrence network analyses and topic modelling (Latent Dirichlet Allocation, LDA) were not normalized using this approach, as they rely on different analytical frameworks. Co-occurrence networks represent relational patterns between terms, where edge weights correspond to the frequency of joint appearances. Similarly, LDA outputs are inherently probabilistic, reflecting normalized topic-term distributions. Therefore, additional normalization was not required for these analyses. Recommendations and their justificatory texts were analysed separately but maintained the country-level structure for all subsequent analyses. In addition, the frequency of occurrence of specific terms—dairy, milk, yogurt, and calcium—was quantified. These specific terms were predefined a priori as core lexical indicators of dairy-products content, representing general product references (dairy, milk), processed variants (yogurt), and key nutrient framing (calcium). All recommendations were transcribed into an Excel database together with the corresponding normalized word frequencies (per 1000 tokens). The resulting dataset was subsequently linked to country-level indicators obtained from the World Bank and the Global Obesity Observatory.

### Text translation and preprocessing for natural language analysis

All analyses were conducted in a Google Colab environment using Python. Prior to natural language processing (NLP) analyses, the dataset was translated into English to ensure linguistic consistency and enable the use of standardized NLP tools. Automated translation was performed using the deep-translator library interfacing with Google Translate. Translated texts were reviewed by the research team to ensure semantic coherence prior to preprocessing and analysis. Following translation, all textual variables were preprocessed using a structured pipeline consistent with standard natural language processing approaches,^22^ while the income_group variable was excluded from transformation. Missing values were removed. Text was normalized by converting all characters to lowercase and removing punctuation, numerical characters, special symbols, and excess whitespace. To address terminological variability, synonyms and lexical variants of dairy products were grouped into standardized categories. This harmonisation process improved semantic consistency and comparability across documents in frequency and co-occurrence analyses.

An extended stopword removal strategy was applied, including standard English stopwords and a curated list of functional words and discourse markers (e.g., articles, auxiliary verbs, and pronouns) that do not contribute to semantic interpretation (Supplementary Table S2). Tokens shorter than three characters were excluded. Lexical harmonisation was applied to ensure semantic consistency across documents, including the unification of orthographic variants (e.g., yoghurt and yogurt) and simple morphological forms (e.g., plural and singular variants) (Supplementary Table S2). To further ensure corpus standardization and comparability across countries, a uniform preprocessing pipeline was applied, including translation into English, lowercasing, removal of punctuation and stopwords, and lexical normalization of equivalent terms. Additionally, word frequencies were normalized and expressed as relative frequencies per 1000 tokens to account for differences in corpus size across income groups.

Lemmatization was then performed using the WordNet lemmatizer, which reduces inflected word forms to their canonical base form (e.g., “products” to “product”) to improve lexical consistency. Word frequencies were subsequently normalized per 1000 tokens to account for differences in corpus size across income groups. This preprocessing approach was applied consistently across all NLP analyses, including frequency analysis, co-occurrence networks, and topic modelling.

### Additional natural language processing analyses

To further characterize the semantic structure of dairy-products dietary messages, complementary NLP analyses were conducted, focusing on lexical associations and latent thematic patterns within recommendations and their justifications. These analyses were designed to extend the descriptive frequency-based results and explore higher-order semantic organization across income groups, without redefining the primary analytical framework. Given the exploratory nature of computational text analysis, results were interpreted as descriptive and comparative representations of semantic patterns, rather than as formal inferential statistical tests. The use of normalized frequencies (per 1000 tokens), consistent preprocessing pipelines, and explicitly defined analytical parameters enhances comparability across countries and reduces bias associated with corpus size and structural heterogeneity. Methods such as lexical frequency analysis, co-occurrence networks, and latent topic modelling are commonly used to explore thematic structures and linguistic patterns in large textual corpora.^23^^,^^24^ Therefore, the analyses were interpreted as descriptive and comparative representations of policy language rather than as hypothesis-testing procedures. Although the analysis focuses on textual data, this study does not follow a traditional qualitative approach based on manual coding or thematic interpretation. Instead, it applies quantitative natural language processing techniques to identify and compare lexical and semantic patterns across documents in a standardized and reproducible manner.

### Lexical co-occurrence network analysis

Word co-occurrence networks were constructed separately for dietary recommendations and justification texts, stratified by World Bank income group (low, lower-middle, upper-middle, and high income). After preprocessing, texts were tokenized (i.e., segmented into individual word units) for computational analysis and filtered to retain the most frequent and semantically relevant terms within each income group. Co-occurrence relationships were defined at the document level, where two terms were considered to co-occur if they appeared within the same country-level guideline section. No fixed sliding window was applied, as the unit of analysis corresponded to the full document. Pairwise co-occurrence matrices were computed based on word presence within the same textual unit (country-level guideline section).^22^ Networks were represented as undirected weighted graphs, where nodes correspond to lexical items and edge weights reflect the absolute frequency of co-occurrence between term pairs. Network properties were interpreted using standard graph-theoretical concepts.^24^ In this context, centrality refers to the relative importance of a node within the network, typically reflecting the number and strength of its connections with other terms. Network density and connectivity were interpreted descriptively as indicators of the overall level of lexical interconnection within each income group, with denser networks reflecting more complex and interconnected semantic structures. To enhance interpretability and reduce network sparsity, only the most frequent terms (top-ranking terms) and their strongest associations were retained. No additional normalization measures (e.g., pointwise mutual information or log-likelihood) were applied, as the analysis aimed to provide a descriptive representation of lexical associations within the corpus. For visualization, only top-ranking terms and strongest associations were retained. Networks were displayed using force-directed layouts, where more strongly connected terms are positioned closer together. Nodes represent individual terms and edges represent co-occurrence relationships between terms within the same document. Edge thickness is proportional to the frequency of co-occurrence, while node size reflects term frequency or centrality within the network.

### Topic modelling analysis

Latent thematic structures within dietary recommendations and justifications were explored using Latent Dirichlet Allocation (LDA). Topic modelling was applied separately to recommendation and justification texts, stratified by income group. In simple terms, LDA identifies groups of words that tend to appear together across documents, allowing these word clusters to be interpreted as underlying topics within the text. All texts underwent the standardized preprocessing pipeline described above, including normalization, extended stopword removal, and lemmatization. Additional domain-specific stopwords (generic connectors, instructional verbs, and temporal qualifiers) were removed to enhance thematic coherence. For each income group, a document–term matrix was constructed separately for recommendation and justification texts. A fixed number of three topics was used for each income group and text type (K = 3 for high-income, upper-middle-income, lower-middle-income, and low-income groups) to maintain comparability across strata and avoid overfitting in relatively small stratified corpora. The number of topics was selected a priori based on parsimony, interpretability, and vocabulary richness after preprocessing, rather than being optimized using coherence score or perplexity. Dictionary filtering retained terms appearing in at least two documents and in no more than 80% of documents within each income group (no_below = 2; no_above = 0.8). LDA models were fitted using gensim.models.LdaModel with 25 passes through the corpus and a fixed random seed (random_state = 42). The document–topic prior was left at the gensim default symmetric setting (α = symmetric), and the topic–word prior was left at the default setting (β/η = default). The maximum number of iterations was also left at the gensim default value of 50. For visualization, the six highest-weighted terms per topic were extracted, and topic relevance was summarized by averaging posterior topic–term probabilities across documents within each income group. No income groups met the exclusion criteria for topic modelling after preprocessing. Topic modelling was implemented using LDA, a probabilistic model widely used to identify latent thematic structures in textual corpora.^25^ Topic relevance was summarized by calculating the mean posterior topic–term probability across documents within each income group. Topic modelling results were treated as exploratory and confirmatory, intended to support and contextualize previously observed lexical patterns rather than to define new primary outcomes. Topic modelling using LDA was specifically employed to capture latent thematic structures that cannot be identified through frequency-based approaches alone, enabling the characterization of higher-order semantic organization across dietary guideline texts.

### Visualisation and reproducibility

All analyses and visualisations were implemented in a reproducible Python-based workflow. All scripts used for text preprocessing and analysis were version-controlled and are available from the authors upon reasonable request. Data manipulation and preprocessing were conducted using pandas, natural language processing relied on nltk and spaCy, network analyses were performed using networkx, and topic modelling was implemented with gensim. Dimensionality reduction and clustering procedures were supported by scikit-learn. Visualisations were generated using matplotlib and seaborn, with consistent colour palettes and scaling applied across income groups. Geographic visualisations were produced using Natural Earth shapefiles, with countries harmonized via ISO 3166-1 alpha-3 codes; Antarctica and sub-national entities were excluded.

### Software and libraries

All analyses were conducted in a reproducible Python-based environment using Google Colab. Data handling and preprocessing were performed with pandas, text translation with deep-translator, and NLP tasks—including stopword removal and lemmatization—were implemented using nltk and spaCy. Progress monitoring during large-scale text processing was facilitated using tqdm.

### Ethics statement

Ethical approval was not required for this study because it involved the analysis of publicly available policy documents and did not include human participants or individual-level data.

### Role of the funding source

No funding was received, and the funding source had no role in the study design, data collection, analysis, interpretation, or writing of the manuscript.

## Results

**Fig. 1:**
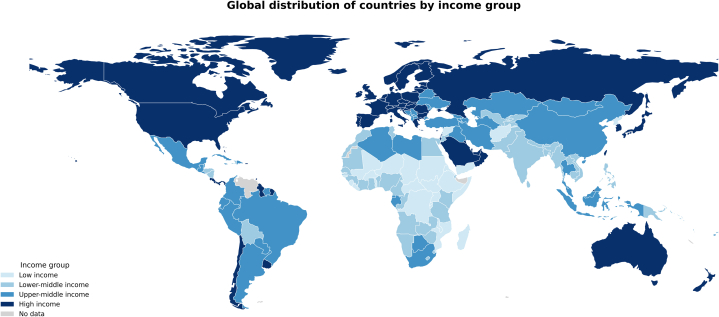
Global distribution of countries by income group. Countries are classified according to the World Bank income group categories (low, lower-middle, upper-middle, and high income). Countries shown in grey indicate territories with no available data after harmonisation. Antarctica and sub-national entities were excluded from the visualization.

**Supplementary Fig. S1:**
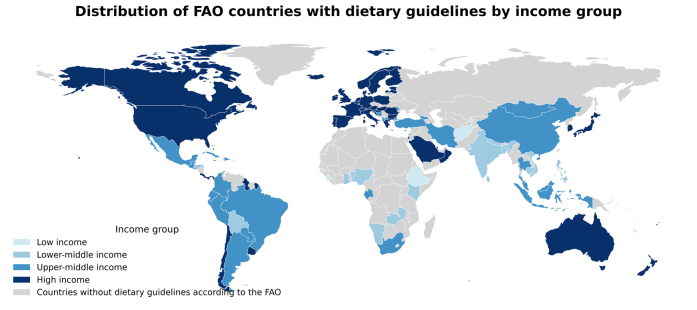


The results are presented within a global socioeconomic context, with countries classified according to World Bank income groups to characterize the economic diversity of the dataset. This classification provides a descriptive framework for interpreting subsequent analyses, while accounting for regional variation and data availability across countries (Fig. 1). Countries with dietary guidelines are predominantly concentrated in high- and upper-middle-income groups, particularly across Europe, North America, Oceania, and parts of East Asia and Latin America. In contrast, representation among low- and lower-middle-income countries is more limited and geographically dispersed, with notable gaps across large areas of sub-Saharan Africa and Central Asia. Countries shown in grey correspond to those without FAO-recognized dietary guidelines, highlighting substantial disparities in the global availability of national dietary guidance across income groups. It should be noted that the FAO repository may not fully reflect the most recent updates for all countries. Notably, the Americas encompass high-, upper-middle-, and lower-middle-income countries, representing all major income categories in which the most pronounced semantic differences were identified. This socioeconomic diversity makes the region a particularly informative setting for understanding how dairy-related dietary messaging varies across development levels (Supplementary Fig. S1).

**Fig. 2:**
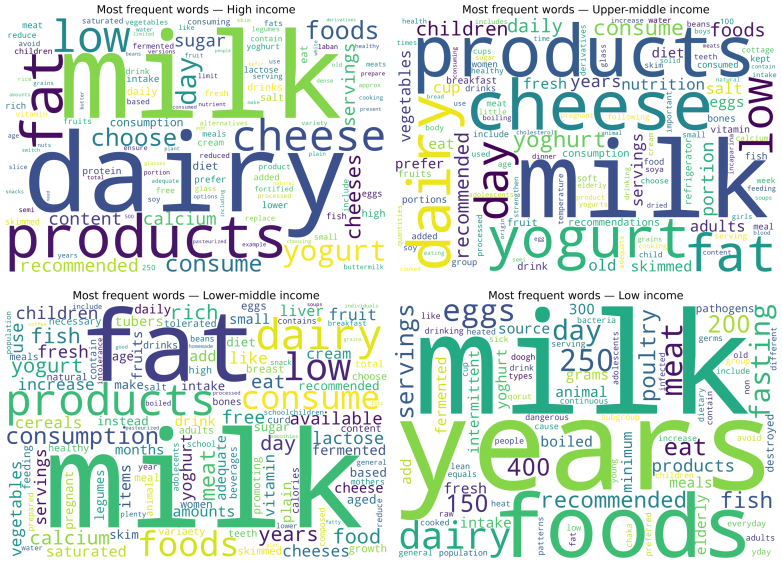
Most frequent words in dairy-related sections of national food-based dietary guidelines stratified by country income group. Word clouds are based on normalized frequencies (per 1000 tokens) to account for differences in corpus size across groups. Text preprocessing included lowercasing, removal of punctuation, and exclusion of English stopwords and short tokens. The size of each word reflects its relative frequency within each income group, with larger words indicating higher prominence in the textual corpus. Differences across panels illustrate variations in the semantic framing of dairy-related recommendations according to socioeconomic context.

Using natural language processing techniques, we analysed the relative frequency and prominence of terms appearing in dairy-related dietary recommendation texts across income groups (Fig. 2). Word frequencies were normalized and expressed as relative frequencies per 1000 tokens to account for differences in corpus size between groups. Across all income groups, “milk” consistently appears as one of the most prominent terms, reflecting its central role in dietary guidance. In high- and upper-middle-income countries, the lexical patterns show a more diversified and product-specific terminology, including frequent references to “cheese”, “yogurt”, and “low-fat”, suggesting a more detailed framing of dairy consumption. In contrast, lower-middle- and low-income countries exhibit a more general and context-oriented language, with greater prominence of terms such as “foods”, “consume”, and “available”. Additionally, the co-occurrence of terms related to other animal-source foods, such as “eggs” and “meat”, indicates that dairy is often presented within a broader nutritional context rather than as an isolated category.

Overall, even after normalization, these patterns highlight meaningful differences in the framing of dairy-related recommendations across socioeconomic contexts. Additionally, some terms referring to specific population groups (e.g., “children”, “adults”, and age-related expressions such as “years”) were observed across income groups. However, these terms did not represent dominant lexical patterns and were not analysed as a separate dimension, as the primary focus of this study was on overall semantic structures in dairy-related messaging (Fig. 2).

**Fig. 3:**
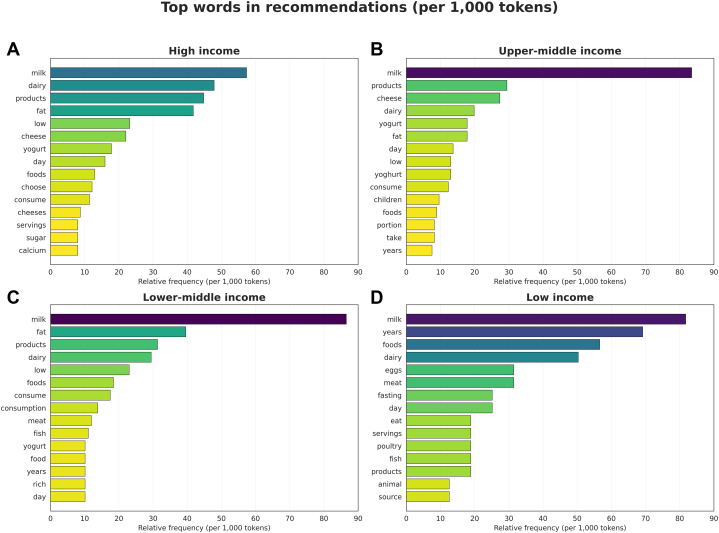
Most frequent words identified in dietary recommendations stratified by income group. The figure shows the top 15 most frequently occurring words extracted from dietary recommendation texts, stratified by income group: High income (A), Upper-middle income (B), Lower-middle income (C), and Low income (D). Bars represent relative word frequencies (per 1000 tokens) within each income group, allowing comparability across corpora of different sizes.

Across all income levels, *milk* consistently emerged as the most frequent term, serving as a common lexical anchor in dairy-products recommendations. However, the composition and relative prominence of accompanying terms varied by income group. In high- and upper-middle-income countries, frequent terms extended beyond *milk* to include *product*, *fat*, *dairy*, *cheese*, and *yogurt*, reflecting more differentiated dietary framing. In contrast, lower-middle- and low-income countries showed a more limited and general vocabulary, with higher relative frequencies of broad food-related terms such as *food*, *meat*, *egg*, *fish*, and temporal or portion-related terms. These patterns are consistent with both the qualitative distribution of terms and the quantitative ranking of the most frequent words across income groups (Fig. 3).

**Fig. 4:**
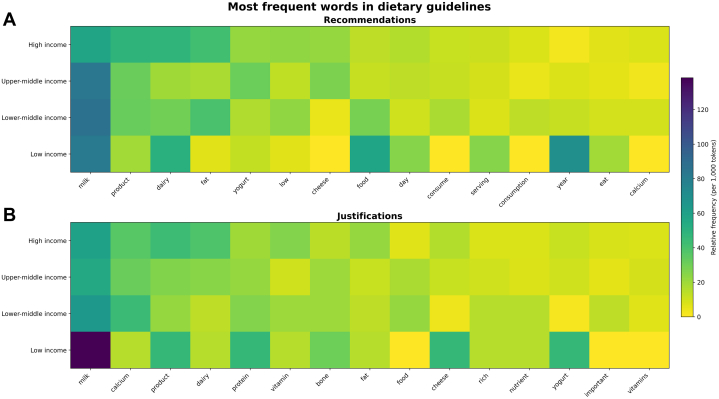
Heatmaps of the most frequent words in dietary guideline texts stratified by income group. The figure presents heatmaps showing the frequency distribution of the most frequent words identified in dietary guideline texts, stratified by income group (High income, Upper-middle income, Lower-middle income, and Low income). Panel (A) displays the most frequent words extracted from dietary recommendations, while panel (B) shows the most frequent words extracted from dietary guideline justifications. Rows correspond to income groups and columns represent the top words identified across the full corpus. Colour intensity reflects relative frequency (per 1000 tokens) within each income group These patterns suggest a clear socioeconomic gradient in the lexical complexity and nutritional framing of dairy-products dietary messages, with progressively narrower thematic scope observed in lower-income settings.

The most frequent words identified in dietary guideline texts showed distinct distribution patterns across income groups when comparing recommendations and their justifications. In dietary recommendations, high-income countries exhibited the highest relative frequencies for terms such as milk, product, fat, and dairy, followed by yogurt, low, and cheese. Upper-middle-income countries also showed a predominance of milk, together with product, fat, dairy, and yogurt, while terms such as consumption, serving, and day appeared with moderate relative frequencies. In lower-middle-income countries, the most frequent recommendation-related terms included milk, product, fat, dairy, and yogurt, whereas words such as consumption, serving, and day were less prominent. In low-income countries, a lower relative prominence of most terms was observed overall, overall word frequencies were lower with milk, food, dairy, year, and meat emerging as the most frequent terms. All heatmaps were generated after lexical normalization and stopword harmonisation, and word frequencies were normalized per 1000 tokens to ensure comparability across income groups. Therefore, the observed differences reflect variations in relative lexical emphasis rather than differences in corpus size or text length (Fig. 4).

In dietary guideline justifications, a similar stratification by income group was observed, although the relative prominence of specific terms differed from recommendations. In high-income countries, justification texts most frequently included milk, calcium, product, vitamin, and dairy, alongside terms such as protein, source, bone, and fat. Upper-middle-income countries showed higher frequencies for milk, calcium, product, vitamin, and protein, with additional recurrent terms including source and fat. In lower-middle-income countries, the most frequent justification-related words were milk, calcium, product, vitamin, and protein, followed by source and bone. In contrast, low-income countries displayed a lower relative prominence of terms overall, with milk, product, fat, and dairy appearing as the most common terms, while nutrient-related words occurred less frequently (Fig. 4). Notably, justification texts across all income groups showed a stronger emphasis on nutrient-related terminology compared with recommendation texts, highlighting their distinct communicative roles within dietary guidelines.

**Supplementary Fig. S2:**
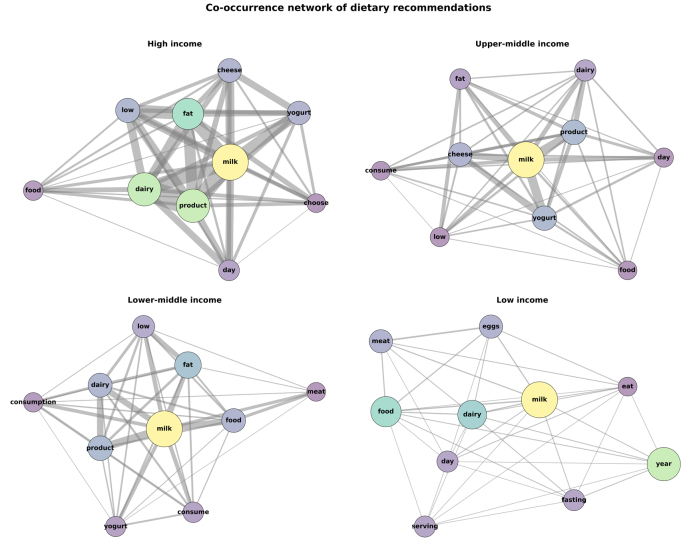


**Supplementary Fig. S3:**
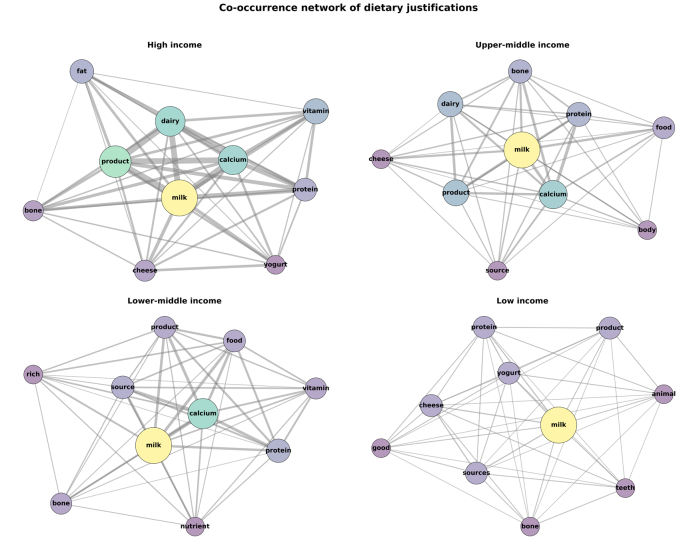


To further explore the internal structure of dietary messaging, co-occurrence networks were constructed separately for recommendations and their justifications across income groups. In these network visualisations, nodes represent individual terms, node size reflects their relative importance within the corpus, and edges represent co-occurrence relationships between terms within the same document. Edge thickness is proportional to the frequency of co-occurrence, with thicker lines indicating stronger associations between terms. These networks visually illustrate the relational organization of key concepts and highlight differences in lexical density and thematic connectivity between income levels. Although treated as exploratory, the co-occurrence networks provide structural support for the frequency-based analyses by illustrating differences in lexical density, centrality, and thematic connectivity across income groups. These visual patterns allow the identification of central terms and tightly connected clusters, which reflect the dominant semantic structures within each income group. After lexical normalization and stopword harmonisation, networks revealed more coherent semantic structures, with high-income groups displaying denser and more differentiated concept clusters, and lower-income groups showing simpler and more centralized lexical patterns (Supplementary Figs. S2 and S3).

**Supplementary Fig. S4:**
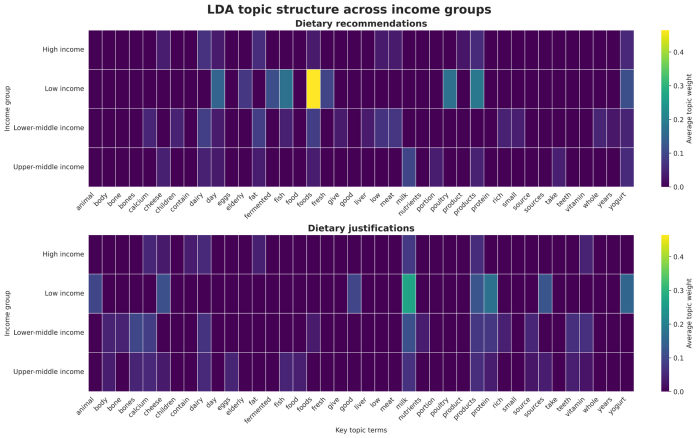


To further explore latent thematic structures beyond frequency-based patterns, topic modelling using Latent Dirichlet Allocation (LDA) was applied to both recommendations and justifications after lexical normalization. These exploratory analyses supported previously observed differences in thematic concentration and lexical emphasis across income groups, without introducing additional primary findings. Given the exploratory nature of topic modelling and the independent estimation of LDA models within each income group, topics were not interpreted as directly comparable across groups. Instead, LDA results were used to contextualize and corroborate previously observed lexical and semantic patterns derived from frequency analyses and co-occurrence networks (Supplementary Fig. S4).

## Discussion

The use of standardized NLP metrics and parameters, including normalized lexical frequencies and consistent preprocessing procedures, enhances the comparability of findings across heterogeneous textual corpora. The present study provides novel evidence on how dairy-products recommendations in FBDGs systematically differ according to countries’ socioeconomic status, using NLP tools. However, it is important to note that the use of country-level classifications may not capture within-country heterogeneity, and therefore these findings should be interpreted as reflecting macro-level patterns that may oversimplify intra-country variability. The findings indicate the presence of a clear semantic gradient, whereby countries with higher income levels and HDI scores tend to present more specific, complex, and diversified messages, while low- and middle-income countries tend to employ more general language focused on basic consumption and the fulfillment of essential nutritional needs.

This pattern is consistent with previous literature that has documented structural heterogeneity in the content of dietary guidelines at the global level.^11^^,^^12^ Comparative studies have shown that dietary guidelines may reflect not only scientific evidence but also political priorities, institutional capacities, national food systems, and specific epidemiological contexts. For example, Herforth et al.,^12^ demonstrated that high-income countries tend to incorporate more detailed messages related to dietary quality, food processing, and chronic disease prevention, whereas lower-income countries prioritize caloric adequacy and the prevention of nutritional deficiencies. Our semantic findings are consistent with this observation from a quantitative linguistic perspective.

The centrality of the term “milk” observed across all income groups suggests that milk continues to serve as the primary conceptual anchor of the dairy food group at a global level. However, the lexical expansion observed in high-income countries—including references to yogurt, cheese, fat, low-fat, and product— may indicate a transition toward more differentiated recommendations, aligned with the contemporary concept of the “dairy matrix”.^26^^,^^27^ Recent research has emphasized that the metabolic effects of dairy products do not depend solely on fat or calcium content, but rather on the structural interaction among nutrients, fermentation processes, and food processing.^8^^,^^28^^,^^29^ This evolving evidence base has been associated with a greater emphasis on fermented products and specific formulations in dietary guidelines from high-income countries.

In contrast, the more restricted vocabulary identified in dairy-products recommendations from low- and middle-income countries suggests a communication approach oriented toward universal and easily understandable messages, possibly related to limitations in nutrition literacy, educational resources, and the diversity of available food products.^11^^,^^30^ This finding is consistent with previous analyses indicating that, in contexts of food insecurity, dietary guidelines tend to prioritize messages focused on basic access to foods that are key sources of protein and critical micronutrients, such as calcium and vitamin B12,^31^ rather than differentiated recommendations based on specific product types.

A relevant aspect is the greater semantic complexity observed in justificatory texts compared with direct recommendations. In high-income countries, justificatory sections incorporated terms associated with specific nutrients (calcium, protein, vitamin), bone health, and metabolism, reflecting a higher degree of integration of scientific evidence into institutional communication.^11^ This finding is consistent with studies showing that dietary guidelines in developed countries tend to include explicit nutritional rationales to support their recommendations, thereby strengthening their technical grounding and clarity.

From a public health perspective, these semantic differences may have relevant implications. The growing global burden of non-communicable chronic diseases, such as type 2 diabetes, obesity, and cardiovascular disease, has been associated with a re-evaluation of the role of dairy products within healthy dietary patterns.^32^, ^33^, ^34^, ^35^, ^36^, ^37^ Recent meta-analyses have shown that dairy consumption—particularly fermented products such as yogurt—is associated with a lower risk of type 2 diabetes and cardiovascular events, while low-fat dairy products may contribute to improved lipid profiles.^38^, ^39^, ^40^ However, more recent studies suggest that the saturated fat content of dairy products may have neutral or even beneficial effects on various health outcomes.^32^^,^^37^ The greater incorporation of such nuances in the dietary guidelines of high-income countries may suggest a more rapid adaptation to emerging scientific evidence.

In contrast, the lower specificity observed in lower-income countries may reflect structural limitations in incorporating complex scientific updates into normative policy documents, as well as logistical constraints related to food availability, cold chain infrastructure, and food safety.^38^^,^^39^ Studies have documented that in many low-income countries, milk and dairy products continue to represent a significant source of foodborne illnesses due to deficiencies in pasteurization, refrigeration, and sanitary control systems.^40^^,^^41^ In this context, it is understandable that dietary guidelines prioritize general and cautious messages, avoiding the promotion of specific dairy subgroups that may not be widely available or safe for consumption.

Another relevant observation is the presence of references to environmental sustainability in dietary guidelines from countries with very high Human Development Index (HDI) levels.^42^, ^43^, ^44^ Although this pattern was identified qualitatively during the review of the documents, it was not formally quantified in the present analysis and should therefore be interpreted with caution. This observation is consistent with recent systematic reviews showing that only a minority of food-based dietary guidelines explicitly incorporate sustainability criteria, despite the growing evidence on the environmental impact of food systems. Several European countries and nations in Oceania have begun to integrate recommendations related to carbon footprint reduction, responsible consumption, and predominantly plant-based dietary patterns with a strategic inclusion of animal-source foods. The more limited presence of this discourse in lower-income countries may reflect immediate priorities related to food security and basic nutrition, rather than a lack of conceptual awareness or interest in sustainability.

These findings should also be interpreted in the context of broader nutritional challenges in many low- and middle-income countries. The coexistence of undernutrition, micronutrient deficiencies, and rising overweight and obesity—commonly described as the “triple burden of malnutrition”—has been widely documented during ongoing nutrition transitions.^18^^,^^45^ In such contexts, dietary guidelines often prioritize ensuring basic nutritional adequacy and food security. In parallel, international organizations such as FAO and WHO have proposed guiding principles for sustainable healthy diets, which aim to promote health while minimizing environmental impacts and ensuring cultural and economic feasibility.^46^^,^^47^

The regional implications of these findings may be particularly relevant for the Americas, especially Latin America and the Caribbean, where countries with markedly different socioeconomic conditions coexist alongside rapid nutrition transitions and the persistent triple burden of malnutrition.^48^ In this context, FBDGs must balance the prevention of nutrient deficiencies with chronic disease prevention and sustainability goals. Regional organizations such as PAHO/WHO may therefore play an important role in supporting greater harmonisation of dietary guidance across the region while considering local socioeconomic and cultural realities.^19^

From a methodological perspective, the use of NLP enables the reproducible, objective, and scalable evaluation of large volumes of textual data. Unlike traditional qualitative analyses, the techniques employed allow for the reproducible, objective, and scalable evaluation of large volumes of text. The analysis of lexical frequencies, co-occurrence networks, and latent topic models enabled the identification of structural patterns that are not always evident through manual review. In particular, the use of topic modelling (LDA) allowed the identification of latent thematic structures beyond surface-level lexical patterns, providing additional insight into the underlying organization of dietary messaging across socioeconomic contexts. Recent studies have highlighted the potential of NLP for the analysis of public health policies, the monitoring of regulatory frameworks, and the comparative evaluation of normative documents, and has increasingly been applied to the analysis of public health policies, regulatory frameworks, and nutrition-related document. Beyond methodological advantages, this approach may also support policymakers in identifying structural disparities in dietary messaging and monitoring the evolution of recommendations across socioeconomic contexts.

Nevertheless, several limitations should be considered when interpreting these findings. Although normalization procedures (relative frequencies per 1000 tokens) were applied to account for differences in corpus size, document length, and vocabulary richness, cross-country comparisons may still be influenced by heterogeneity in document structure, level of detail, and country representation within each income group. In addition, although all documents were translated into English and processed using a standardized natural language processing (NLP) pipeline, contextual factors such as geographic region, cultural context, publication year, and guideline development processes were not formally controlled for and may have influenced the linguistic patterns identified. Consequently, country income classification may act as a proxy for broader structural and contextual differences rather than an independent explanatory factor. The analytical approach was primarily descriptive and exploratory, and no inferential statistical models were applied to formally test differences between groups. Furthermore, the study focused exclusively on the content and framing of dietary guidelines and did not incorporate population-level dairy consumption data, preventing assessment of whether the observed linguistic differences correspond to actual dietary behaviours. The corpus was also limited to dietary guidelines available through the FAO platform, and variability in document completeness and depth—particularly among lower-income countries, where fewer guidelines were available—may have influenced the representation of dairy-related content. Finally, while NLP techniques enable reproducible, objective, and scalable analyses of large textual corpora, they may not fully capture the nuance, contextual meaning, or policy intent underlying specific recommendations. Therefore, the observed semantic differences should be interpreted as indicators of variation in dietary messaging rather than as direct measures of policy quality, implementation, or effectiveness. Future research integrating contextual variables, food-system characteristics, and quantitative dietary data may help further clarify the implications of these findings for nutrition policy and population health.

Despite these limitations, the results have implications for the future design of food and nutrition policies. The evidence suggests that moving toward more specific, culturally adapted guidelines aligned with emerging scientific evidence may improve communicative effectiveness, particularly in contexts of nutritional transition. Furthermore, an NLP-based approach could be leveraged by international organizations to monitor the temporal evolution of dietary recommendations and to assess convergence or divergence across regions. Although a standardized preprocessing pipeline was applied and all texts were translated into English to ensure linguistic consistency, other contextual factors such as geographic region, year of publication, and country-specific policy frameworks may also influence the framing of dietary recommendations. An additional limitation relates to the representation of low-income countries. Many low-income countries do not have formally established food-based dietary guidelines, and when such guidelines are available, they are often shorter, less detailed, and lack fully developed recommendation and justification sections. This structural disparity may have influenced the observed differences in lexical complexity and semantic patterns, and should be interpreted as reflecting underlying inequalities in policy development capacity rather than solely differences in dietary messaging.

### Conclusions

This study demonstrates systematic structural and semantic differences in dairy-products recommendations within national dietary guidelines, closely associated with countries’ levels of socioeconomic development. By applying natural language processing techniques, we identified linguistic patterns that reflect differences in contextual conditions, public policy priorities, and institutional capacity.

These findings suggest that socioeconomic context should be considered when formulating and updating dietary guidelines, and indicate that advanced computational approaches can support comparative analyses of nutrition policies and evidence-informed public health strategies. However, these differences should be interpreted within the broader context of cultural dietary traditions, food system structures, and agricultural practices, which may also shape how dietary recommendations are framed. These findings may be particularly relevant for the Americas, where countries with diverse socioeconomic realities coexist and where PAHO/WHO have promoted efforts toward evidence-based and regionally harmonized dietary guidance. Understanding how dairy-related messages differ across development levels may contribute to future discussions on nutrition policy harmonisation while preserving cultural and contextual relevance.

Future research should extend these findings by examining the relationship between dietary guideline language and real-world dietary behaviours, as well as evaluating the implementation and effectiveness of recommendations across diverse socioeconomic contexts. The observed variability in dairy-related messaging may have implications for the dairy sector, potentially influencing product development, reformulation strategies, and market positioning in response to evolving nutritional guidance. From a policy and practice perspective, these findings may support governments in identifying gaps in guideline specificity, guide the food sector in aligning product communication with public health messaging, and inform future research on the relationship between policy language and dietary behaviours. Overall, NLP-based approaches provide a scalable and reproducible framework for the comparative evaluation of dietary guidelines and nutrition policy documents.

## Contributors

AB: conceptualization, data curation, formal analysis, methodology, visualization, writing – original draft, and writing – review & editing.; HC-O: data curation, formal analysis conceptualization, visualization, writing – original draft, and writing – review & editing.; SD-A: Conceptualization, investigation, methodology, and supervision. RV: Investigation and visualization; RV investigation, visualization, writing – original draft, and writing – review & editing. All authors had full access to the data, read and approved the final version of the manuscript.

## Data sharing statement

Deidentified processed textual datasets and analytical scripts generated during this study are available from the corresponding author upon reasonable request for academic research purposes.

## Editor note

The Lancet Group takes a neutral position with respect to territorial claims in published maps and institutional affiliations.

## AI use statement

ChatGPT (OpenAI, GPT-5.5) was used solely to improve the clarity, readability, and English language of the manuscript. All content was reviewed, edited, and approved by the authors, who take full responsibility for the accuracy and integrity of the work.

## Declaration of interests

The authors declare no competing interests.

## Appendix A Supplementary data

Supplementary Table S2

Supplementary captions
