# Supplementary material for: Global and regional inequalities in dairy recommendations: a natural language processing analysis of food-based dietary guidelines across income groups
Source: Lancet Reg Health Am. 2026 Jul 6;62:101550. doi: 10.1016/j.lana.2026.101550 (PMC13355785; doi:10.1016/j.lana.2026.101550)
Supplement: Supplementary Table S1 [file mmc1.docx]

**Supplementary Table 1. Summary of dairy recommendations and justifications by country.**

| **income_group** | **Country** | **Year** | **Number of recomendations** | **Number of justifications** |
| --- | --- | --- | --- | --- |
| High income | Antigua and Barbuda | 2013 | 0 | 0 |
| High income | Australia | 2013 | 8 | 2 |
| High income | Austria | 2010 | 2 | 0 |
| High income | Bahamas | 2002 | 0 | 0 |
| High income | Barbados | 2017 | 1 | 0 |
| High income | Belgium | 2019 | 6 | 1 |
| High income | Bulgaria | 2006 | 10 | 1 |
| High income | Canada | 2019 | 0 | 0 |
| High income | Chile | 2022 | 7 | 2 |
| High income | Costa rica | 2011 | 6 | 1 |
| High income | Croatia | 2018 | 1 | 1 |
| High income | Cyprus | 2007 | 1 | 0 |
| High income | Denmark | 2021 | 9 | 3 |
| High income | Estonia | 2025 | 7 | 2 |
| High income | Finland | 2019 | 10 | 4 |
| High income | France | 2019 | 7 | 0 |
| High income | Germany | 2024 | 1 | 1 |
| High income | Greece | 2014 | 17 | 0 |
| High income | Guyana | 2018 | 0 | 0 |
| High income | Hungary | 2016 | 3 | 0 |
| High income | Iceland | 2024 | 1 | 0 |
| High income | Ireland | 2012 | 5 | 1 |
| High income | Israel | 2008 | 1 | 0 |
| High income | Italy | 2018 | 2 | 1 |
| High income | Japan | 2000 | 1 | 0 |
| High income | Latvia | 2020 | 4 | 2 |
| High income | Malta | 2016 | 6 | 0 |
| High income | Netherlands | 2015 | 1 | 0 |
| High income | New zealand | 2020 | 1 | 0 |
| High income | Norway | 2024 | 6 | 3 |
| High income | Oman | 2009 | 7 | 8 |
| High income | Panama | 2013 | 1 | 2 |
| High income | Poland | 2020 | 5 | 0 |
| High income | Portugal | 2016 | 2 | 0 |
| High income | Qatar | 2015 | 18 | 1 |
| High income | Romania | 2006 | 5 | 1 |
| High income | Saint Kitts and Nevis | 2010 | 22 | 0 |
| High income | Saudi Arabia | 2012 | 3 | 2 |
| High income | Seychelles | 2006 | 1 | 0 |
| High income | Slovenia | 2015 | 2 | 0 |
| High income | South Korea | 2015 | 2 | 0 |
| High income | Spain | 2022 | 2 | 1 |
| High income | Sweden | 2015 | 5 | 4 |
| High income | Switzerland | 2020 | 3 | 2 |
| High income | United Kingdom | 2018 | 5 | 0 |
| High income | United States | 2026 | 9 | 2 |
| High income | Uruguay | 2016 | 4 | 3 |
| Upper-middle income | Albania | 2008 | 16 | 4 |
| Upper-middle income | Argentina | 2018 | 4 | 1 |
| Upper-middle income | Belize | 2012 | 0 | 3 |
| Upper-middle income | Bosnia and Herzegovina | 2004 | 0 | 0 |
| Upper-middle income | Brazil | 2015 | 2 | 1 |
| Upper-middle income | China | 2022 | 1 | 1 |
| Upper-middle income | Colombia | 2019 | 7 | 2 |
| Upper-middle income | Cuba | 2009 | 2 | 3 |
| Upper-middle income | Dominica | 2007 | 0 | 0 |
| Upper-middle income | Dominican Republic | 2009 | 1 | 0 |
| Upper-middle income | Ecuador | 2020 | 1 | 1 |
| Upper-middle income | Fiji | 2013 | 3 | 3 |
| Upper-middle income | Gabon | 2021 | 1 | 0 |
| Upper-middle income | Georgia | 2005 | 3 | 1 |
| Upper-middle income | Grenada | 2020 | 0 | 0 |
| Upper-middle income | Guatemala | 2012 | 5 | 1 |
| Upper-middle income | Indonesia | 2014 | 1 | 0 |
| Upper-middle income | Iran | 2015 | 1 | 0 |
| Upper-middle income | Jamaica | 2015 | 1 | 0 |
| Upper-middle income | Malaysia | 2010 | 2 | 0 |
| Upper-middle income | Mexico | 2023 | 0 | 0 |
| Upper-middle income | Moldova | 2019 | 5 | 5 |
| Upper-middle income | Mongolia | 2010 | 2 | 0 |
| Upper-middle income | North Macedonia | 2014 | 2 | 0 |
| Upper-middle income | Paraguay | 2015 | 11 | 3 |
| Upper-middle income | Peru | 2020 | 2 | 1 |
| Upper-middle income | Saint Lucia | 2007 | 0 | 0 |
| Upper-middle income | Saint Vincent and the Grenadines | 2021 | 1 | 0 |
| Upper-middle income | South Africa | 2013 | 4 | 7 |
| Upper-middle income | Thailand | 1998 | 4 | 1 |
| Upper-middle income | El Salvador | 2012 | 5 | 2 |
| Upper-middle income | Turkey | 2006 | 18 | 2 |
| Lower-middle income | Bangladesh | 2013 | 4 | 1 |
| Lower-middle income | Benin | 2015 | 1 | 1 |
| Lower-middle income | Bolivia | 2013-2014 | 13 | 4 |
| Lower-middle income | Cambodia | 2017 | 3 | 3 |
| Lower-middle income | Ghana | 2023 | 1 | 0 |
| Lower-middle income | Honduras | 2013 | 2 | 1 |
| Lower-middle income | India | 2011 | 11 | 10 |
| Lower-middle income | Kenya | 2017 | 9 | 1 |
| Lower-middle income | Lebanon | 2013 | 22 | 5 |
| Lower-middle income | Namibia | 2000 | 0 | 0 |
| Lower-middle income | Nepal | 2012 | 1 | 0 |
| Lower-middle income | Nigeria | 2006 | 15 | 0 |
| Lower-middle income | Philippines | 2012 | 1 | 0 |
| Lower-middle income | Sri Lanka | 2020 | 1 | 0 |
| Lower-middle income | Vietnam | 2020 | 1 | 0 |
| Lower-middle income | Zambia | 2021 | 10 | 3 |
| Low income | Afghanistan | 2016 | 2 | 2 |
| Low income | Ethiopia | 2022 | 5 | 2 |
| Low income | Sierra Leone | 2016 | 3 | 0 |

PAG: It only indicates a website, but the year is not specified.

A recommendation aims to guide the consumption of a food group; it may include recommended portions, examples of foods belonging to that group, culinary preparations, etc.

Example of a recommendation: “Consume dairy at all stages of life.”

A nutritional justification explains the reason behind the recommendations; it may include health benefits or risks, bioactive components, etc.

Example of a nutritional justification: “Milk and dairy products are the only important sources of vitamin B12 in the nutrition of lactovegetarians.”
