# Supplementary material for: Global and regional inequalities in dairy recommendations: a natural language processing analysis of food-based dietary guidelines across income groups
Source: Lancet Reg Health Am. 2026 Jul 6;62:101550. doi: 10.1016/j.lana.2026.101550 (PMC13355785; doi:10.1016/j.lana.2026.101550)
Supplement: Supplementary Table S2 [file mmc2.docx]

**Table S2. Curated stopwords and lexical harmonization rules used in text preprocessing**

**A. Curated stopwords**

| **Stopwords** |
| --- |
| the |
| and |
| or |
| of |
| to |
| in |
| on |
| for |
| from |
| by |
| with |
| that |
| this |
| these |
| those |
| your |
| should |
| such |
| other |
| use |
| using |
| recommended |
| recommend |
| daily |
| are |
| not |
| which |
| they |
| can |

**B. Lexical harmonization rules**

| **Original term** | **Standardized term** |
| --- | --- |
| yoghurt | yogurt |
| proteins | protein |
| products | product |
| foods | food |
| bones | bone |
| nutrients | nutrient |
| years | year |
| servings | serving |

*Note: All preprocessing steps were applied consistently across the full corpus, including lowercasing, punctuation removal, stopword filtering, and lexical harmonization, to ensure comparability across income groups.*
