# Supplementary material for: Global and regional inequalities in dairy recommendations: a natural language processing analysis of food-based dietary guidelines across income groups
Source: Lancet Reg Health Am. 2026 Jul 6;62:101550. doi: 10.1016/j.lana.2026.101550 (PMC13355785; doi:10.1016/j.lana.2026.101550)
Supplement: Supplementary captions [file mmc3.docx]

**Supplementary material**: The online appendix contains Supplementary Figures S1–S4 and tables S1–S2.

**Supplementary Figure 1.** **Distribution of FAO countries with dietary guidelines by income group**. Countries with FAO-recognized dietary guidelines are shown according to World Bank income group classification (low, lower-middle, upper-middle, and high income). Countries displayed in grey indicate the absence of national dietary guidelines recognized by the FAO. Antarctica was excluded from the visualization.

**Supplementary Figure 2**. Co-occurrence networks of dairy-products recommendations texts by income group.

**Supplementary Figure 3**. Co-occurrence networks of dairy-products justifications texts by income group.

**Supplementary Figure 4**. Latent Dirichlet Allocation (LDA) topics of dietary recommendations and justifications by income group

**Supplementary Table 1.** Summary of dairy recommendations and justifications by country.

**Supplementary Table 2.** Curated stopwords and lexical harmonization rules used in text preprocessing.
